# *Eoalosa janvieri* gen. et sp. nov., a new clupeid fish (Teleostei, Clupeiformes) from the Eocene of Monte Bolca, Italy

**DOI:** 10.1007/s12542-017-0378-0

**Published:** 2017-08-18

**Authors:** Giuseppe Marramà, Giorgio Carnevale

**Affiliations:** 1 Department of Palaeontology, University of Vienna, Geozentrum, Althanstraβe 14, 1090 Vienna, Austria; 2 Dipartimento di Scienze Della Terra, Università degli Studi di Torino, Via Valperga Caluso, 35 10125 Turin, Italy

**Keywords:** *Eoalosa janvieri* gen. et sp. nov., Clupeidae, Palaeobiodiversity, Konservat-Lagerstätte, Ypresian, *Eoalosa janvieri* gen. et sp. nov., Clupeidae, Paläobiodiversität, Konservat-Lagerstätte, Ypresium

## Abstract

Fishes of the family Clupeidae are extremely abundant in the Eocene fossiliferous limestone of Monte Bolca representing the most common group from this celebrated locality. A new clupeid from the Pesciara site, *Eoalosa janvieri* gen. et sp. nov., is described. The new taxon exhibits a unique combination of characters supporting its recognition as a new genus and species of clupeid fish that is tentatively placed in the subfamily Alosinae. The description of this new taxon improves our knowledge of the diversity of clupeoid fishes in the Eocene of Monte Bolca.

## Kurzfassung

Fische aus der Familie der Clupeidae sind sehr häufig in den fossilführenden eozänen Schichten von Monte Bolca anzutreffen und repräsentieren die häufigste Knochenfischgruppe in dieser berühmten Fundstelle. Ein neuer Clupeide aus Monte Bolca, *Eoalosa janvieri* gen. et sp. nov., wird hier beschrieben und diskutiert. Dieses neue Taxon zeigt eine einzigartige Kombination von Merkmalen, welche die Aufstellung einer neuen Gattung und Art innerhalb der Unterfamilie Alosinae erlaubt. Die Beschreibung dieses neuen Heringsartigen trägt wesentlich zur Kenntnis der Diversität clupeoider Fische im Eozän von Monte Bolca bei.

## Introduction

Fishes of the suborder Clupeoidei (herrings, sardines, shads, sprats, round herrings, and anchovies) represent one of the most abundant and widespread groups of teleosts. They currently comprise 91 genera and more than 400 extant species (Nelson et al. 2016) arranged in four families (Chirocentridae, Clupeidae, Engraulidae, Pristigasteridae), with a fossil record dating back to the Early Cretaceous (Figueiredo 2009). The suborder Clupeoidei includes small- to medium-sized fishes with a worldwide distribution from tropical to cold-temperate waters, where they form large schools that feed mainly on zooplankton (Whitehead 1985). Diagnostic characters of the Clupeoidei include, amongst others, the fusion of the first uroneural with the first preural centrum, a reduction in the relative size of the first ural centrum, an autogenous parhypural, loss of the lateral line scales, a single row of gill rakers on arches one to three, and second and third infraphryngobranchials that are reduced to long, narrow processes anteriorly (Grande 1985; Nelson et al. 2016). Fishes of the family Clupeidae differ from other clupeoids by having two long rod-like postcleithra in the pectoral girdle (Grande 1985). Clupeids have a moderately elongate and fusiform body, a mouth that is usually terminal (inferior in Dorosomatinae), teeth that are small or absent, a short-based dorsal fin located at the midlength of the body, pelvic fins placed just in front of, below, or just behind the dorsal-fin base, and a short anal fin that originates well behind the last dorsal-fin ray (Whitehead 1985).

The celebrated Eocene (ca. 50 Ma) Konservat-Lagerstätte of Monte Bolca is one of the most famous and well-studied palaeontological sites in the world. It has yielded a huge amount of exquisitely preserved fishes that have provided clear evidence of the highly successful Early Palaeogene fish radiation in the aftermath of the extinction event at the end of the Cretaceous (Marramà et al. 2016a, b, c). More than 230 species (Carnevale et al. 2014)—largely anguilliforms, aulopiforms, atheriniforms, beryciforms, clupeiforms, lophiiforms, pleuronectiforms, tetraodontiforms, and several other percomorph groups (e.g., Blot 1969, 1978; Blot and Tyler 1990; Tyler and Santini 2002; Bannikov 2004a, b, 2006, 2008; Monsch 2006; Bannikov and Carnevale 2009, 2010, 2016; Carnevale and Pietsch 2009, 2010, 2011, 2012; Marramà and Carnevale 2015a, b, 2016, 2017)—have been described to date. In particular, clupeiforms are represented by three clupeoid taxa: the sardine *Bolcaichthys catopygopterus*, the round herring *Trollichthys bolcensis*, and the engraulid *Eoengraulis fasoloi*. A recent taphonomic and quantitative palaeoecological study (Marramà et al. 2016a) revealed that the fish-bearing strata of the two productive sites of Monte Bolca, the Pesciara and Monte Postale, originated under different environmental conditions, and that the clupeid *Bolcaichthys catopygopterus* represents the most common taxon in the Pesciara fish assemblage in terms of number of specimens.

The aim of this work—another in a series of papers focused on the Eocene clupeoid fishes from Monte Bolca (see Marramà and Carnevale 2015a, b, 2016)—is to describe a new genus and species of clupeid fish based on a single specimen that was recently discovered in the palaeoichthyological collection of the Museum National d’Histoire Naturelle, Paris (MNHN).

## Materials and methods

The present study is based on a unique well-preserved specimen with a preservation quality and slab lithology that clearly indicate it was collected at the Pesciara site. The specimen (MNHN F.Bol475) was examined using a Leica M80 stereomicroscope equipped with camera lucida drawing arm. Alcohol was used to enhance some details of the skeletal anatomy. Measurements were taken using a dial caliper to the nearest 0.1 mm. The standard length (SL) is used throughout. Osteological terminology primarily follows Ridewood (1904), Phillips (1942), Grande (1985), and Marramà and Carnevale (2015a, b). Comparative information was derived mainly from the literature.

### Systematic palaeontology

Superorder **Clupeomorpha** Greenwood, Rosen, Weitzman and Myers, 1966

Order **Clupeiformes** Bleeker, 1859

Suborder **Clupeoidei** Bleeker, 1859

Family **Clupeidae** Cuvier, 1817

Genus ***Eoalosa*** gen. nov.

*Type species*. *Eoalosa janvieri* sp. nov.

*Etymology*. The name is derived from the Greek word *Ēōs* (meaning “dawn,” “sunrise”), and the Latin word *Alausa* (meaning “shad”).

*Other species included*. Type species only.

*Diagnosis*. Clupeid fish with elongated and tapered body, herring-like; SL about 3.5 times head length; mouth terminal; gape of the mouth extending posteriorly up to the anterior margin of the eye; two supramaxillae; strong and keeled abdominal scutes (12 prepelvic and at least 5 postpelvic) bearing thick ascending arms; at least 13 supraneurals; 47 vertebrae; 26 pleural ribs; pleural ribs to preural vertebrae ratio about 0.55; three epurals; dorsal fin small, inserting at about the mid-length of the body, containing about 15 rays; anal fin contains about 17 rays; pelvic-fin origin slightly behind the vertical through the origin of the dorsal fin; pelvic fin contains seven rays; scales perforated.

*Remarks*. The Alosinae, commonly known as shads, constitute an assemblage of temperate to tropical marine, estuarine, or freshwater fishes that include some of the largest clupeid species (Whitehead 1985). Traditionally, alosines include seven living genera (*Alosa*, *Brevoortia*, *Ethmalosa*, *Ethmidium*, *Gudusia*, *Hilsa*, and *Tenualosa*) and about 42 species (Nelson et al. 2016). However, only four genera (*Alosa*, *Brevoortia*, *Sardinops*, and *Sardina*) were recognized in this subfamily by Lavoué et al. (2014) based on molecular data. *Caspialosa* and *Pomolobus*, traditionally included within the alosines, are currently regarded as junior synonyms of *Alosa* (Whitehead 1985; Faria et al. 2006). Although the Alosinae, as well as other clupeid subfamilies, have long been considered ‘groups of convenience’ (Grande 1985) and were found to be non-monophyletic in molecular studies (e.g., Lavoué et al. 2007, 2014), several authors recognized a set of morphological characters that were useful for separating alosines from other clupeid genera, including their large size (up to 60 cm in SL), the possession of strong and well-developed abdominal scutes, and the presence of a median notch between the two contralateral premaxillae, plus some features of the gill arches (e.g., basihyal cartilaginous, mediopharyngobranchial always present) and of the digestive tract (e.g., Nelson 1967; Whitehead 1973, 1985; Zaragüeta-Bagils 2001; Baykina and Schwarzhans 2017). As a consequence of this confused interpretative scenario, *Eoalosa janvieri* is tentatively referred herein to the Alosinae, based on a set of morphological characters observed in the examined specimen and discussed below (see “Discussion”).

***Eoalosa janvieri*** sp. nov.

**Fig. 1:**
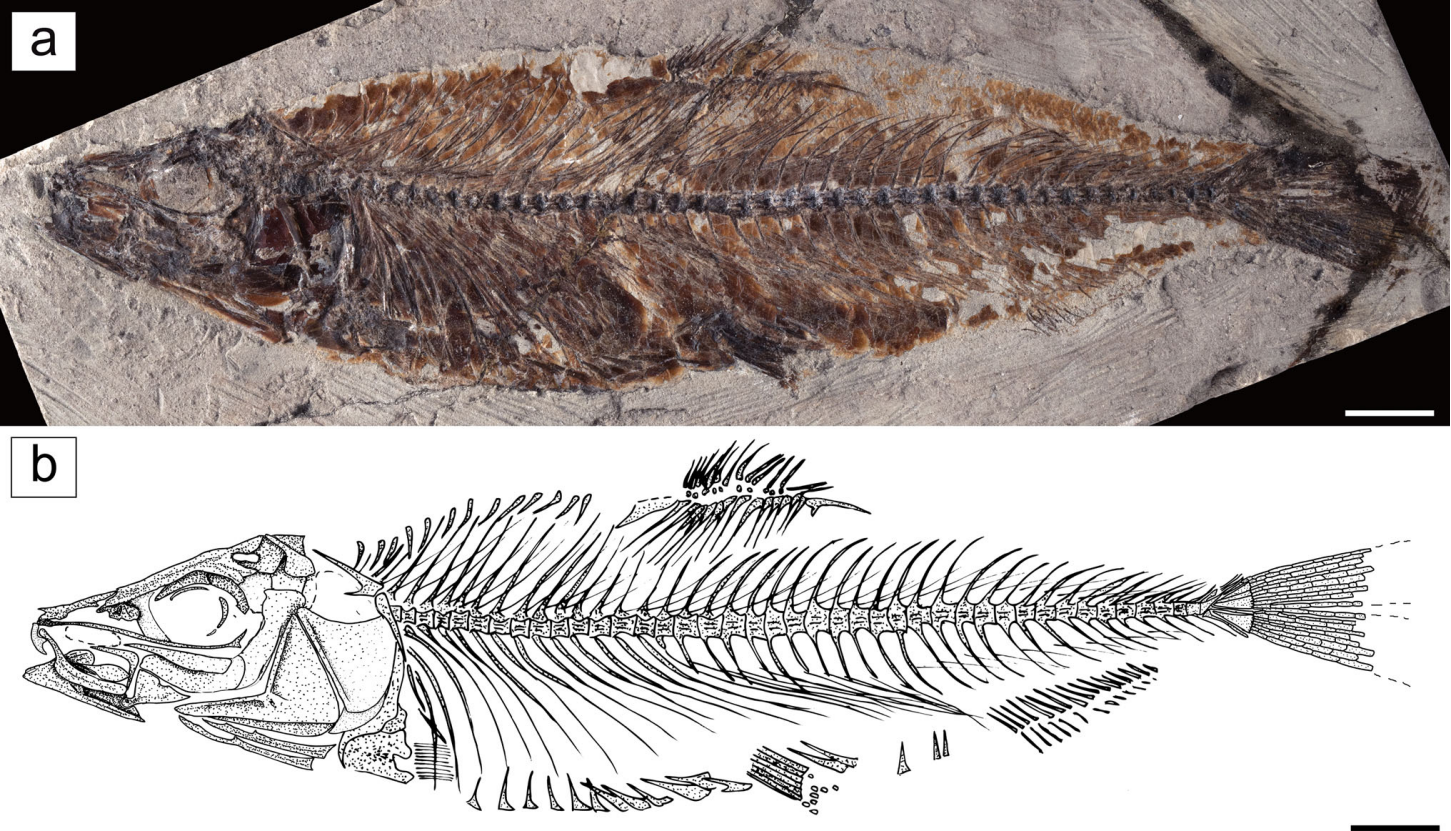
*Eoalosa janvieri* gen. et sp. nov. **a** MNHN F.Bol475, holotype. **b** Reconstruction (scales omitted). *Scale bars* 10 mm

**Fig. 2:**
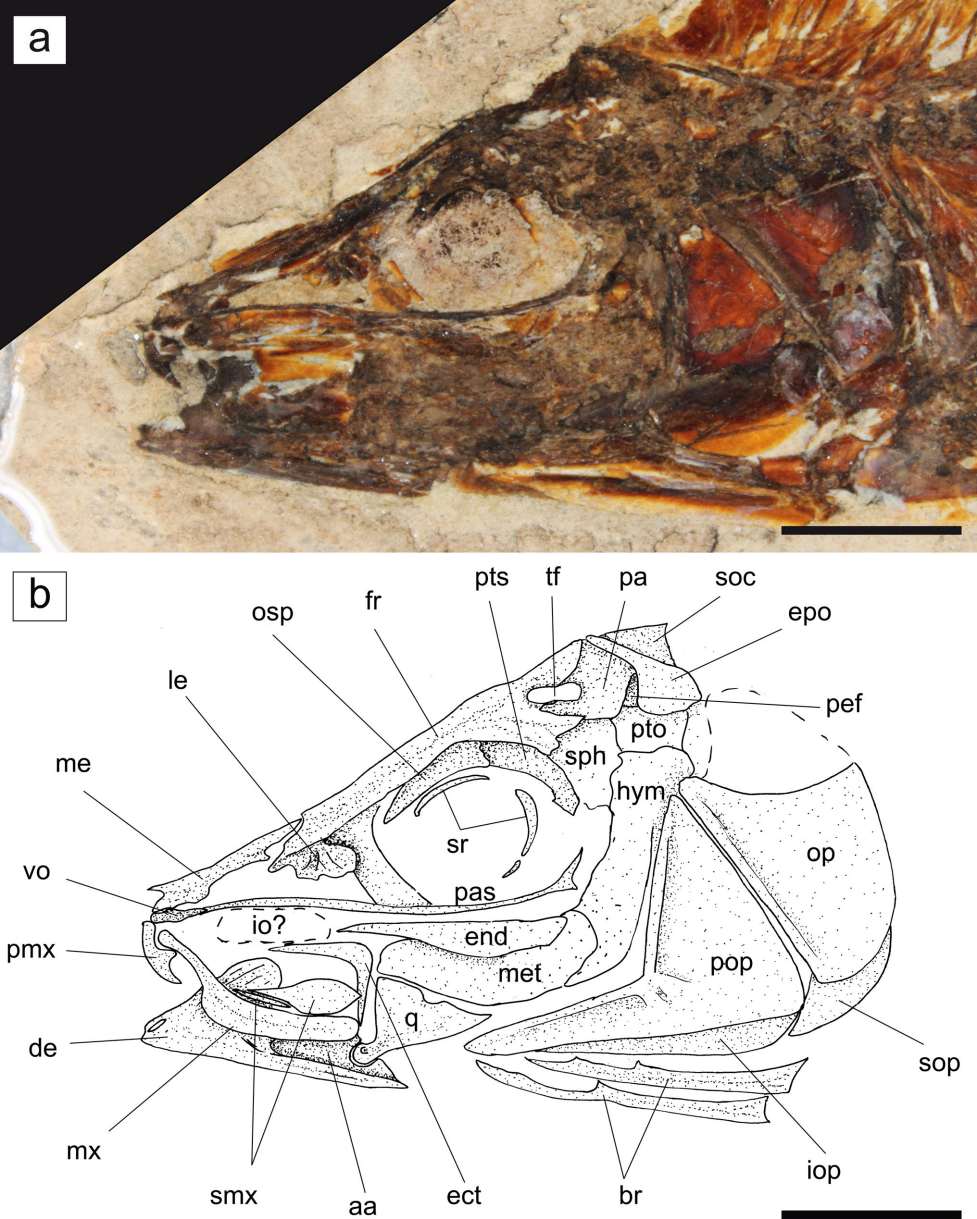
*Eoalosa janvieri* gen. et sp. nov.; MNHN F.Bol475, holotype. **a** Close-up of the head. **b** Reconstruction. *Scale bars* 10 mm. Abbreviations: *aa* anguloarticular, *de* dentary, *ect* ectopterygoid, *end* endopterygoid, *epo* epioccipital, *fr* frontal, *hym* hyomandibula, *io?* infraorbital, *iop* interopercle, *le* lateral ethmoid, *me* mesethmoid, *met* metapterygoid, *mx* maxilla, *op* opercle, *osp* orbitosphenoid, *pa* parietal, *pas* parasphenoid, *pef* pre-epiotic fossa, *pmx* premaxilla, *pop* preopercle, *pto* pterotic, *pts* pterosphenoid, *q* quadrate, *smx* supramaxilla, *soc* supraoccipital, *sop* subopercle, *sph* sphenotic, *sr* sclerotic ring, *tf* temporal foramen, *vo* vomer

**Fig. 3:**
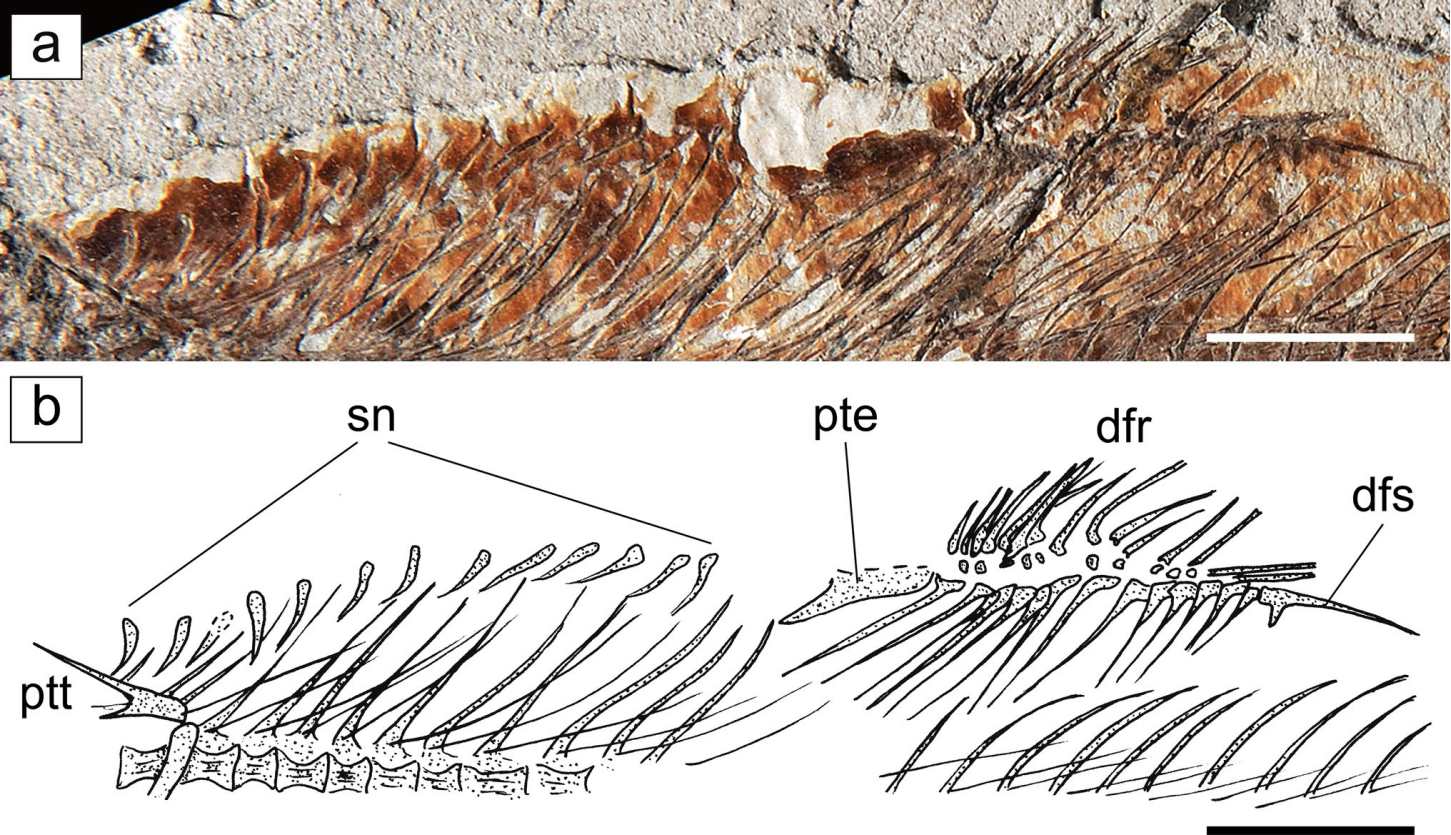
*Eoalosa janvieri* gen. et sp. nov.; MNHN F.Bol475, holotype. **a** Detail of the anterodorsal margin of the body showing the supraneurals and the dorsal fin. **b** Reconstruction (scales omitted). *Scale bars* 10 mm. Abbreviations: *dfr* dorsal-fin rays, *dfs* dorsal-fin stay, *pte* first dorsal-fin pterygiophore, *ptt* posttemporal, *sn* supraneurals

**Fig. 4:**
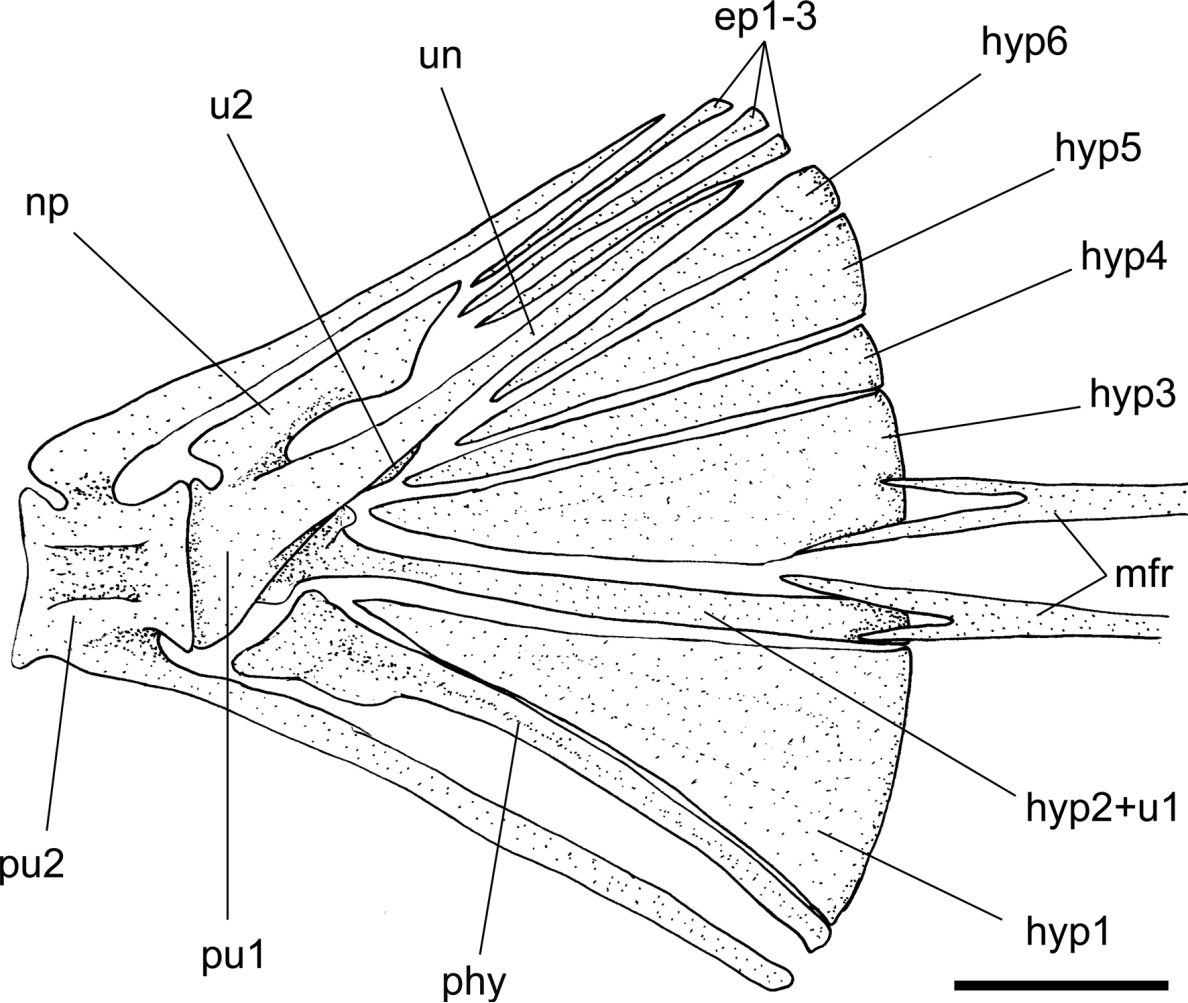
*Eoalosa janvieri* gen. et sp. nov.; MNHN F.Bol475, holotype; reconstruction of the caudal skeleton. *Scale bar* 2 mm. Abbreviations: *ep* epural, *hyp* hypural, *mfr* median caudal-fin rays, *np* neural plate, *phy* parhypural, *pu* preural centrum, *u* ural centrum, *un* uroneural

**Fig. 5:**
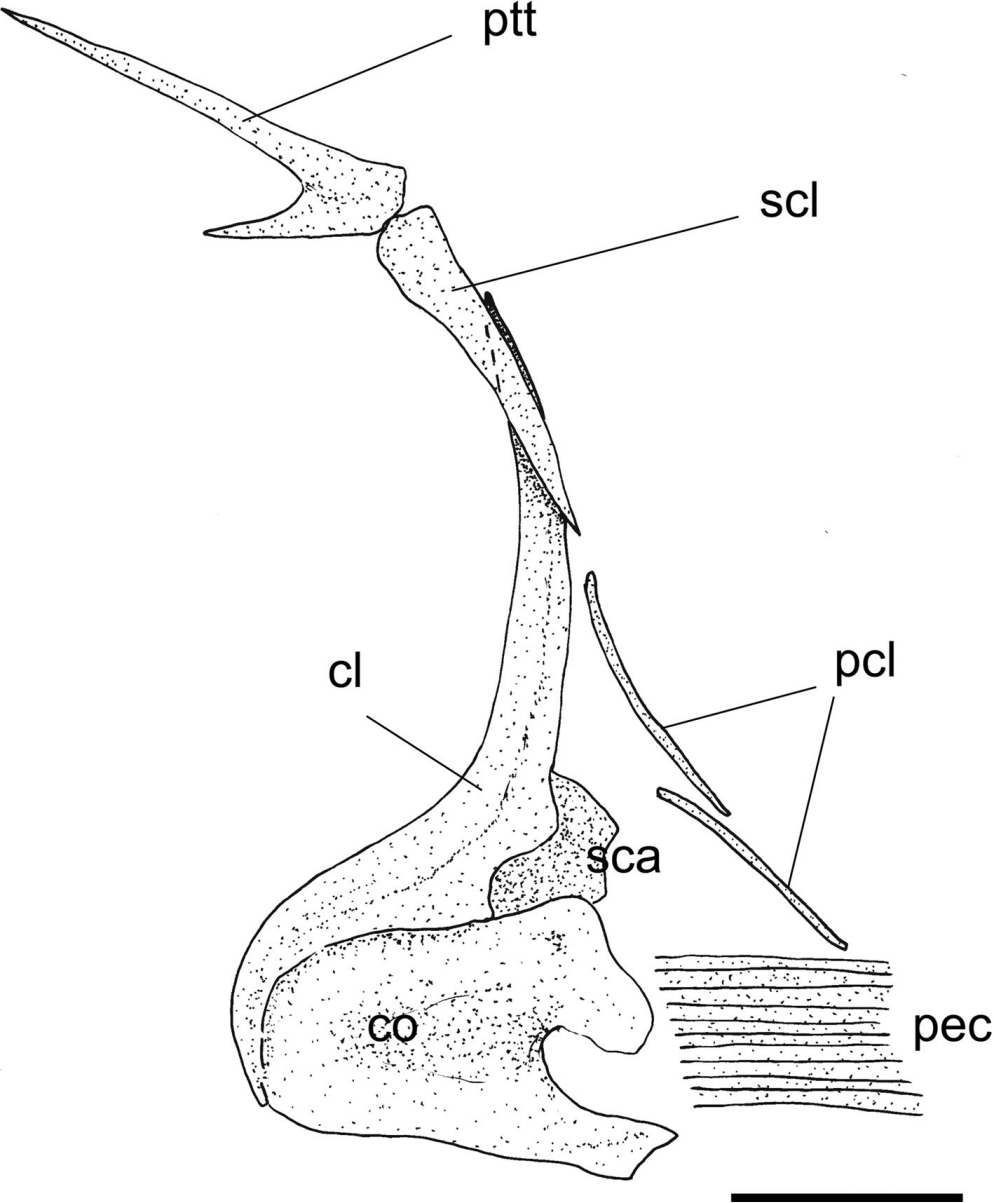
*Eoalosa janvieri* gen. et sp. nov.; MNHN F.Bol475, holotype; reconstruction of the pectoral girdle. *Scale bar* 5 mm. Abbreviations: *cl* cleithrum, *co* coracoid, *pcl* postcleithra, *pec* pectoral-fin rays, *ptt* posttemporal, *sca* scapula, *scl* supracleithrum

**Fig. 6:**
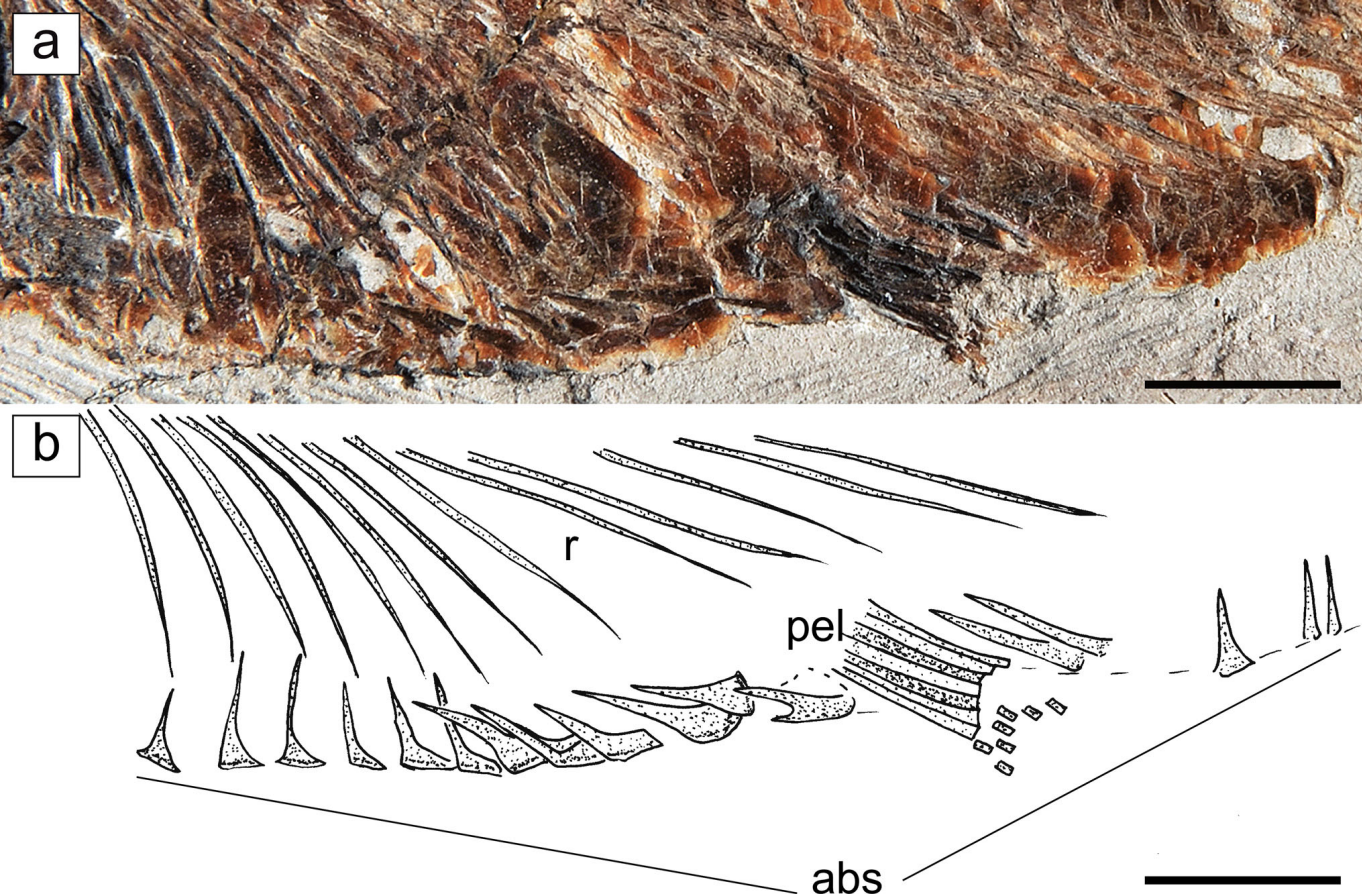
*Eoalosa janvieri* gen. et sp. nov.; MNHN F.Bol475, holotype. **a** Detail of the abdominal region showing the abdominal scutes. **b** Reconstruction (scales omitted). *Scale bars* 10 mm. Abbreviations: *abs* abdominal scutes, *pel* pelvic-fin rays, *r* ribs

**Fig. 7:**
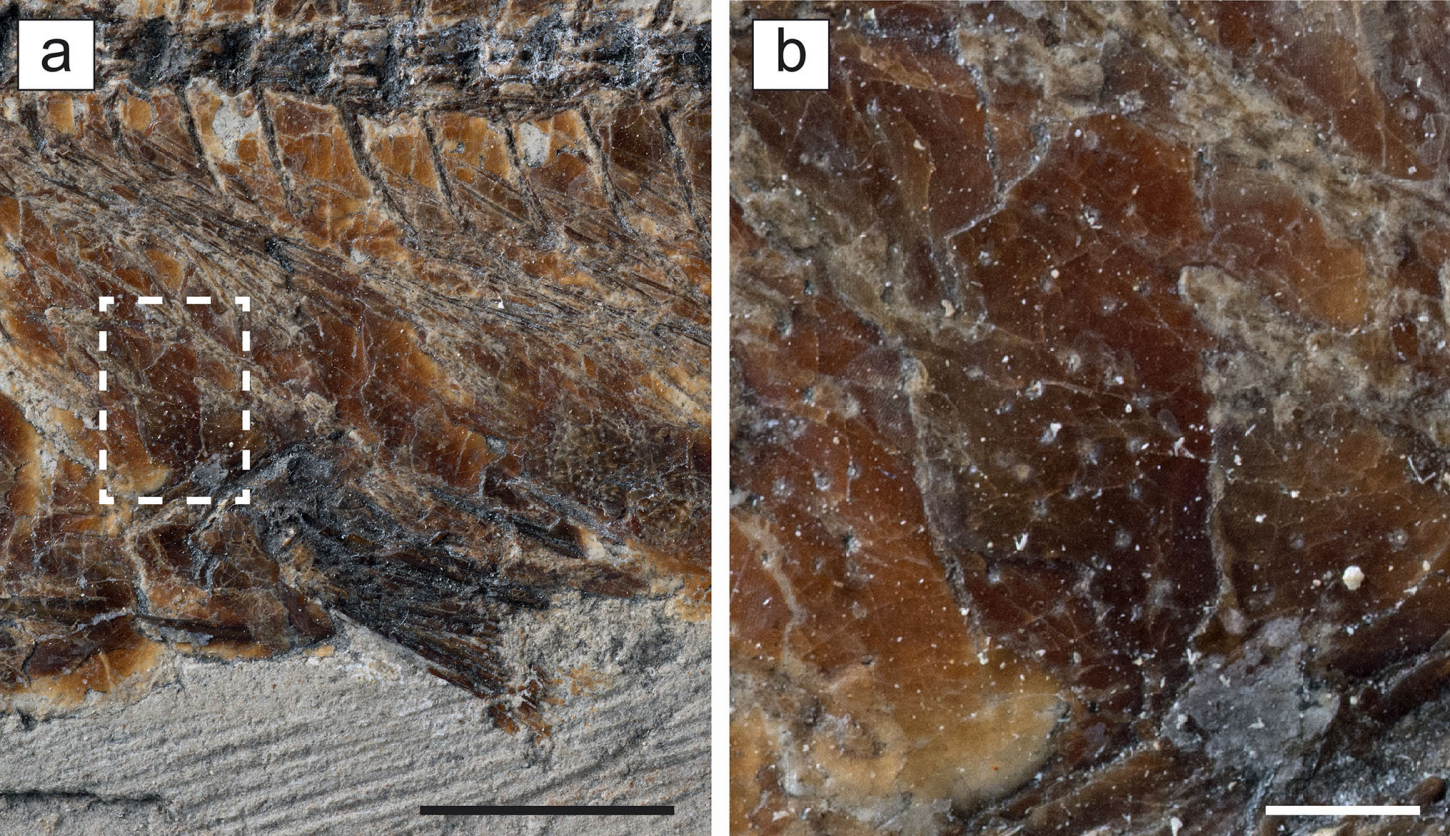
*Eoalosa janvieri* gen. et sp. nov.; MNHN F.Bol475, holotype. **a** Close-up of the scales of the abdominal region, *scale bar* 10 mm. **b** Detail of the area indicated, *scale bar* 1 mm

Figures 1, 2, 3, 4, 5, 6, and 7.

*Etymology*. Named in honour of the distinguished French palaeoichthyologist Philippe Janvier.

*Holotype*. MNHN F.Bol475, well-preserved, near-complete articulated skeleton on a single plate; 155.7 mm SL (Fig. 1).

*Locality and horizon*. Monte Bolca, Pesciara site, NE Italy; late Early Eocene, Late Ypresian, Middle Cuisian, slightly less than 50 Ma (see Papazzoni et al. 2014).

*Diagnosis*. As for the genus.

*Measurements* (in mm; percentage of SL in parentheses). Head length: 43.7 (28.1); head depth: 26.1 (16.8); preorbital length: 12.3 (7.9); postorbital length: 17.1 (11.0); orbit diameter: 10.7 (6.9); maximum body depth: 41.2 (26.5); caudal peduncle length: 17.4 (11.2); caudal peduncle depth: 12.3 (7.9); predorsal length: 80.9 (52.0); preanal length: 125.1 (80.4); prepectoral length: 45.5 (29.2); prepelvic length: 88.9 (55.1); dorsal-fin base length: 17.5 (11.2); anal-fin base length: 14.9 (9.6).

### Description

The body is elongate and laterally compressed, herring-like (Fig. 1); its greatest depth is about one-quarter the SL. The head is moderately elongate and triangular in outline; the SL is about 3.5 times the head length. The snout is pointed. The mouth is small and terminal. The belly is armored with strong and keeled prepelvic and postpelvic abdominal scutes bearing thick ascending arms compared to other clupeids. The dorsal-fin origin is located at about mid-length of the body, whereas the pelvic fins insert slightly behind the vertical through the dorsal-fin origin. The body is completely covered with large and perforated cycloid scales.

*Neurocranium*. The neurocranium is elongate and nearly triangular in lateral outline (Fig. 2). The paired frontals are the largest bones of the skull roof, occupying about 60–70% of the neurocranial length. Each of the frontals articulates anteriorly with the mesethmoid, anteroventrally with the lateral ethmoid, and posteriorly with the parietal. Moreover, they articulate ventrally with the orbitosphenoid, and posteroventrally with the pterosphenoid. It is unclear if frontoparietal striae are present in the specimen examined. The parietal is a subrectangular bone that articulates anteriorly with the frontal, anteroventrally with the sphenotic, posterodorsally with the epioccipital, and posteroventrally with the pterotic. A small, ovoid temporal foramen is demarcated by the posterior margin of the frontal and the anterior margin of the parietal. The pre-epiotic fossa is rather small. The supraoccipital crest is only partially preserved, and appears to be pointed and subtriangular in shape. The epioccipital articulates anteriorly with the parietal and posteriorly with the upper limb of the posttemporal. The otic region is badly preserved and cannot provide any information on its structure and configuration. The orbitosphenoid and pterosphenoid form the dorsal and posterior walls of the orbit, respectively. The basisphenoid is not clearly recognizable. The parasphenoid is very long, thin, and gently curved, extending for the most of the basicranial length. The vomer is short and apparently toothless. The mesethmoid is long, slender, and irregular in outline. The lateral ethmoid is expanded ventrally and bears a few striae along its outer surface.

*Circumorbital series*. The orbital cavity is large and rounded. The diameter of the orbit is similar to the preorbital length (about 7% of the SL), resembling the condition of *Alosa* cf. *crassa* (see Carnevale 2007). Except for the first infraorbital, the bones of the circumorbital series are insufficiently preserved in the available specimen. Two semicircular plates of the sclerotic ring are partially recognizable.

*Jaws*. The edentulous premaxilla is small, robust, and subtriangular in shape. Since the specimen is preserved in lateral view, it is not possible to fully describe the peculiar median notch between the two premaxillae typical of the Alosinae (Whitehead 1985) into which the symphysis of the lower jaw fits. The mouth gape of *Eoalosa* extends posteriorly close to the vertical from the anterior margin of the eye. The maxilla is elongate and laterally flattened, not reaching the anterior margin of the orbit; it is angled in the anterior one-third of its length, with a narrow anterior and an expanded posterior portion forming an angle of about 130°. The oral margin of the maxilla is slightly convex and appears to be edentulous. *Eoalosa* possesses two supramaxillae; the first is thin, straight, and rod-like, whereas the second is large and paddle-shaped. The dentary is robust, deep, and its general morphology is similar to that of *Alosa* (see Svetovidov 1964, fig. 8). The dentary appears to be edentulous and a small flattened ovoid foramen lies along its anteroventral margin. The anguloarticular is robust but its morphology is not clearly recognizable.

*Suspensorium*. The palatine is not recognizable. The ectopterygoid is well developed, forming an angle of about 90° between its dorsal (horizontal) and ventral portions. The endopterygoid is elongate and expanded posteriorly. The metapterygoid is subquadrangular in shape. The quadrate is triangular in shape, and its well-developed condyle of the quadrate-articular joint is located just below the anterior margin of the orbit. There is no trace of the large ovoid foramen between the quadrate and metapterygoid that is characteristic of certain species of *Alosa* (see Ridewood 1904), even if a small notch is visible along the dorsal margin of the quadrate. The hyomandibula is poorly preserved, but it clearly articulates ventrally with the metapterygoid (Fig. 2).

*Opercular series and hyoid bar*. The preopercle is large and crescent-shaped; its vertical arm is slightly shorter than the horizontal one. The number of branches of the preopercular sensory canal is impossible to define. The opercle is broad, with a gently rounded posterior margin and a straight anterior margin. Its surface is apparently smooth, without radiating striae or grooves. The subopercle is small and triangular in shape, whereas the interopercle is relatively long and slender. The hyoid bar and gill arches are not clearly recognizable. Two (or three?) long and robust branchiostegal rays are visible under the ventral arm of the preopercle (Fig. 2), but the precise number of them is difficult to discern. Small projections seem to be present dorsally on each branchiostegal. Since some clupeiforms possess projections ventrally on branchiostegal rays (McAllister 1968), we cannot exclude the possibility that their unusual position in *Eoalosa* might be due to preservation bias.

*Vertebral column and intermuscular bones*. The vertebral column consists of 47 preural vertebrae, including the urostylar element, of which 20 are abdominal and 27 caudal (Fig. 1). The vertebral centra are subquadrangular in shape with a slight constriction in the middle. Neural and hemal arches are well developed throughout the entire length of the vertebral column. The neural prezygapophyses and the neural and hemal postzygapophyses are short, not expanded. 26 pairs of pleural ribs appear to be present, with the first rib articulating with the third vertebra; all ribs reach the ventral margin of the body. The ratio of the number of pleural ribs to the number of preural vertebrae (= pleural ribs–preural vertebrae ratio of Grande 1985) is 0.55, thereby reflecting the relatively large size of the abdominal cavity, which is typical of representatives of the Clupeoidea (see Grande 1985). The epineurals are very long and thin, and extend posteriorly for most of the caudal region; they articulate with the base of the neural spines from the third or fourth vertebra to the 19th or 20th vertebrae, becoming short and free from articulation in the caudal region. Epipleurals and epicentrals are not clearly recognizable, although short epipleurals are visible in the caudal region. There are at least 13 slender and backward-inclined supraneurals, with a dorsal tip that reaches the dorsal border of the body (Fig. 3). However, the presence of a preservational gap in front of the dorsal fin suggests that more supraneurals would have been present.

*Caudal skeleton*. The structure of the caudal skeleton of *Eoalosa* (Fig. 4) is consistent with that of other clupeoid fishes (Grande 1985). The first preural centrum is short and subtriangular in shape; dorsally, it bears a laminar and trapezoidal neural plate. The first ural centrum is fused to the second hypural. The second ural centrum is small. There are six hypurals, all autogenous except for the second one, which is elongate, slender, and fused to the first ural centrum; the first hypural appears to be the largest of the series. The parhypural is autogenous and slender, characterized by an expanded proximal end. The first uroneural is long, rod-like, and fused with the first preural centrum. There is no clear evidence of other uroneurals. There are three slender, straight, and rod-like epurals. The caudal fin contains 19 or 20 principal caudal-fin rays, of which the central two bear a dorsal and a ventral peg, as in all alosines, most clupeines, and the pristigasterid *Ilisha* (Grande 1985). The procurrent rays are not clearly recognizable.

*Median fins.* The dorsal fin originates at about the mid-length of the body, just above the 21st to 27th vertebrae; it is short-based and contains about 15 rays supported by an equal number of pterygiophores. There is a long dorsal-fin stay (sensu Weitzman 1962) (Fig. 3). The anal fin lies well behind the dorsal-fin base; it is short and might contain about 17 rays, based on the presence of an equal number of pterygiophores; the endoskeletal elements of the anal fin appear to be associated with the hemal spines of the 33rd to 38th vertebrae. There are no separate finlets. The anal-fin stay is extremely short.

*Paired fins and girdles*. The pectoral girdle (Fig. 5) is consistent with those of other clupeid fishes (see Grande 1985). The posttemporal is long and bifurcated, with a thin and rod-like dorsal arm. The supracleithrum is long and slightly curved. The cleithrum is the largest element of the pectoral girdle. The coracoid is robust and triangular in outline with a convex anteroventral margin and a nearly concave posterior margin. There are two long and rod-like postcleithra. The scapula is partially hidden by the coracoid. The mesocoracoid and proximal radials are not clearly recognizable. The pectoral fin contains at least ten rays, but the original number of them is probably obfuscated by taphonomic processes. The pelvic fin originates slightly behind the dorsal-fin origin and contains seven distally segmented rays. Due to the thickness of the scales covering the abdominal region, the basipterygia are not clearly exposed and cannot be properly described.

*Abdominal scutes*. *Eoalosa* is characterized by a continuous series of abdominal scutes that extend from the isthmus to the anal-fin origin (Fig. 6). There are 12 prepelvic, one pelvic, and at least five postpelvic keeled scutes bearing thick ascending arms. The abdominal scutes are strong and well ossified, especially those located just in front of the pelvic fins. Considerably well-developed abdominal scutes are regarded as diagnostic of the alosines (e.g., Whitehead 1985; Baykina and Schwarzhans 2017; Nelson et al. 2016), although several other clupeoids show this character (e.g., pristigasterids; see Grande 1985, fig. 11). There are no predorsal scutes.

*Squamation*. The entire body is covered by thick and large cycloid scales (Fig. 1a). The scales are deeply overlapping, well-attached, and arranged in about 30–40 transverse scale rows. The number of horizontal rows is difficult to define. The scales are subcircular in shape and highly perforated along their entire surface (Fig. 7), resembling the condition of certain alosines of the genera *Alosa* and *Hilsa* (e.g., Szymczyk 1978; Whitehead 1985; Munroe et al. 1999). However, in contrast to extant species, the perforations are present both in the anterior and posterior regions of the body. The posterior margin of the scales appears to be smooth. Vertical striae or circuli are not evident on the scales. Lateral line scales are absent.

## Discussion

Descriptive analysis of the skeletal morphology of *Eoalosa janvieri* revealed the presence of several diagnostic characters that unquestionably support its inclusion within the Clupeomorpha, including one or more abdominal scutes crossing the ventral midline of the body, presence of the pre-epiotic fossa, second hypural fused with the first ural centrum, and an autogenous first hypural (Grande 1985). Because of the inadequate preservation of the otic region of the neurocranium, two additional remarkable clupeomorph synapomorphies (an otophysic connection that penetrates the exoccipital and forms ossified bullae in the prootic and usually also in the pterotic; a supratemporal commissural sensory canal that primitively passes through parietals) cannot be checked in the single available specimen described herein. The assignment of *Eoalosa* to the Clupeoidei is justified by the fusion of the first uroneural with the first preural centrum, the reduction of the first ural, the presence of an autogenous parhypural, and the absence of lateral-line scales (Grande 1985). The relatively high pleural rib to preural vertebrae ratio (0.55) suggests that *Eoalosa* is a member of the superfamily Clupeoidea. The assignment to the family Clupeidae is clearly supported by the presence of two long, rod-like postcleithra (Grande 1985).

Grande (1985) and Whitehead (1985) recognized five subfamilies within the Clupeidae (Alosinae, Clupeinae, Dorosomatinae, Dussumieriinae, Pellonulinae), although recent molecular studies suggest that this family might be non-monophyletic (e.g., Lavoué et al. 2007, 2014; Li and Ortí 2007). Because of its unique combination of osteological and meristic features, *Eoalosa* cannot be confidently assigned to any of the previously described clupeid genera (see, e.g., Grande 1985; Whitehead 1985). *Eoalosa* exhibits a series of prepelvic and postpelvic scutes, thereby implying that it cannot be referred to the subfamily Dussumieriinae, whose representatives are characterized by an absence of prepelvic and postpelvic scutes and the presence of a single W-shaped scute immediately anterior to the pelvic fins (Grande 1985; Whitehead 1985). *Eoalosa* cannot be placed within the subfamily Pellonulinae because the representatives of this group are characterized by a missing anterior supramaxilla and the fusion of the first ural centrum with the first preural centrum (Grande 1985). Although there is no unambiguous osteological evidence supporting the monophyletic status of the subfamilies Dorosomatinae, Clupeinae, and Alosinae, any assignment of *Eoalosa* to the Dorosomatinae can be ruled out because of the absence of the features that characterize the genera of this assemblage, including a dentary that is flared outwards, mouth inferior, a snout usually rounded and projecting, and a filamentous last dorsal-fin ray (Grande 1985; Whitehead 1985). Although the morphology of the gill arches may include some derived characters that are useful for separating the Clupeinae from the Alosinae (Nelson 1967; Grande 1985), the inadequate preservation of the gill skeleton does not allow the observation of these structures. According to several studies, the Alosinae can be distinguished from the Clupeinae by the presence of a medium to large or very large body (from 150 to 600 mm in SL), the presence of predorsal scutes (e.g., in *Ethmidium*, *Ethmalosa*, and some species of *Alosa*), an upper jaw frontally not rounded and characterized by a distinct notch into which the symphysis of the lower jaw fits, and the presence of a full range of highly developed abdominal scutes (Svetovidov 1963, 1964; Whitehead 1973, 1985; Baykina and Schwarzhans 2017). Moreover, certain alosines (e.g., *Alosa* cf. *sagorensis* and *Hilsa*) are characterized by body scales that are considerably perforated along their entire surface (Szymczyk 1978; Whitehead 1985; Munroe et al. 1999); clupeine genera usually do not exhibit this character, except for *Sardinella*, which possesses scales that are perforated only in the hind part (Whitehead 1985). *Eoalosa* reaches about 156 mm SL (it probably reached about 200 mm in total length), making it markedly larger than most Clupeinae. *Eoalosa* also possesses strong and well-developed prepelvic and postpelvic scutes and completely perforated body scales. Although some representatives of the Pristigasteridae also possess strong ventral scutes (see Grande 1985, fig. 11), we can exclude the possibility that *Eoalosa* belongs to this family because of an absence of typical pristigasteroid characters (e.g., supraneurals oriented either vertically or inclined anterodorsally, and missing interlobar notch in the third hypural; Grande 1985). For these reasons, *Eoalosa* is tentatively assigned herein to the subfamily Alosinae.

*Eoalosa* differs from the other alosine genera by having a unique combination of meristic features (Table 1). It can be easily separated from *Brevoortia*, *Ethmalosa*, *Ethmidium*, *Gudusia*, *Hilsa*, *Moldavichthys*, and *Pugliaclupea* based on its higher number of supraneurals (at least 13 vs. 6–12). *Eoalosa* lacks the dorsal scutes that are diagnostic of some species of *Alosa* (e.g., *A. aestivalis*), *Ethmalosa*, and *Ethmidium*. The number of preural vertebrae is useful for separating *Eoalosa* (47) from *Ethmalosa*, *Gudusia*, *Hilsa*, *Moldavichthys*, ‘*Pomolobus*’ and *Tenualosa* (39–45), *Ethmidium* (49), and possibly from *Pugliaclupea* (estimated to be 38–40). Moreover, *Eoalosa* has seven pelvic-fin rays, in contrast to *Alosa*, *Moldavichthys*, and ‘*Pomolobus*’ (8–11). It also possesses about 15 dorsal-fin pterygiophores, whereas *Ethmalosa*, *Ethmidium*, *Hilsa*, and *Moldavichthys* have 16–19 elements and *Chasmoclupea* has 12. *Eoalosa* also has about 17 anal-fin pterygiophores, compared with the 18–27 of *Brevoortia*, *Ethmalosa*, *Gudusia*, and *Hilsa* and the 12–15 of *Ethmidium*. Finally, the number of epurals can be used to separate *Eoalosa* (3) from *Brevoortia*, *Ethmidium*, *Hilsa*, *Moldavichthys*, and *Pugliaclupea* (1–2).

**Table 1:** Summary of selected morphological features used to discriminate recent and fossil genera of the subfamily Alosinae

Taxon	Supraneurals	Dorsal scutes	Branchiostegal rays	Pleural ribs (pair)	Preural vertebrae	Pleural ribs–preural vertebrae ratio	Pelvic-fin rays	Dorsal-fin pterygiophores	Anal-fin pterygiophores	Epurals
*Alosa*	9–13	0–1	7–8	26–33	46–57	0.54–0.60	9–11	12–20	15–23	1–3
*Brevoortia*	10–12	0	7	24–27	43–50	0.54–0.56	7	17–24	18–24	1–2
*Chasmoclupea*	13	0	?	17	40 + ?	?	7	12	?	?
*Eoalosa* gen. n.	13 + ?	0	?	26	47	0.55	7	15	17	3
*Ethmalosa*	8	2	6	24	43	0.56	7	16–19	19–23	3
*Ethmidium*	11	24	9	31	49	0.63	7	19	12–15	2
*Gudusia*	8–9	0	6	21	41–42	0.50–0.51	7	15–16	22–27	2–3
*Hilsa*	7	0	6	24	41–45	0.53–0.59	7	16–19	21–23	2
*Moldavichthys*	9–10	0	7–8	20–25	39–44	0.54	8	16–17	17–18	2
‘*Pomolobus*’	?	0	?	?	40–43	?	8–9	14–17	17–22	?
*Pugliaclupea*	6	?	?	19	38–40*	0.50*	7	15	?	1
*Tenualosa*	?	0	6	?	44–45	?	7	14–21	16–23	?

The table includes new data and data from Grande (1985), Whitehead (1985), Munroe (2000), Taverne (2004, 2007), Murray et al. (2005), Baykina and Schwarzhans (2017), Froese and Pauly (2016). ‘*Pomolobus*’ solely includes the species ‘*P*.’ *antiquus*, ‘*P*.’ *curtus*, and ‘*P*.’ *facilis* from the Oligocene of Caucasus described by Danil’chenko (1960); these species should not be referred to *Alosa* because they differ from it in vertebral count

The asterisks (*) indicate the estimated count

As discussed above, three clupeoid genera are known from Monte Bolca, including an engraulid (*Eoengraulis*) and two clupeids (*Bolcaichthys* and *Trollichthys*) (Marramà and Carnevale 2015a, b, 2016). *E. janvieri* is easily distinguishable from *Eoengraulis fasoloi* because of the lack of the diagnostic characters typical of anchovies, including the presence of a prominent snout with the mesethmoid projecting beyond the vomer, and an obliquely inclined suspensorium (Grande and Nelson 1985; Marramà and Carnevale 2016). *Trollichthys bolcensis* is considered the earliest unambiguous round herring (subfamily Dussumieriinae) due to the absence of prepelvic and postpelvic abdominal scutes and the presence of a unique W-shaped pelvic scute (Marramà and Carnevale 2015a), characters which are absent in *Eoalosa janvieri*. The sardine *Bolcaichthys catopygopterus* is by far the most abundant clupeoid in the Eocene fish assemblage of Monte Bolca (Marramà and Carnevale 2015b; Marramà et al. 2016a). *Eoalosa* can be easily separated from this clupeine based on its larger size (the maximum SL for *B. catopygopterus* is about 10 cm), much stronger and well-developed abdominal scutes, and different meristic counts.

The oldest fossil currently assigned to the Alosinae, *Pugliaclupea nolardi* from the Late Cretaceous (Campanian, ca. 74 Ma) of southern Italy (Taverne 2004), was created based on a single incomplete specimen lacking the caudal region. Subsequently, Taverne (2007) described an isolated incomplete caudal skeleton and referred it to the same taxon. *Pugliaclupea* was assigned to the Alosinae because of its overall similarity to the extant alosine genera. The evidence supporting the assignment of this Cretaceous clupeoid to the Alosinae appears to be weak, and new largely complete material would be necessary to demonstrate such a hypothesis. With the exception of *Pugliaclupea nolardi*, all the fossil species that have been referred to the Alosinae come from Cenozoic deposits (see Grande 1985). Following the common practice of most of the authors from the nineteenth and twentieth centuries, most of them have been referred to the basket genus *Alosa* (e.g., Sauvage 1873; Woodward 1901; Arambourg 1927; Rückert-Ülkümen 2006; but see Grande 1985 for a comprehensive list of the fossil species assigned to this genus), although taxonomic revisions would certainly demonstrate that they do not all belong to *Alosa* (Zaragüeta-Bagils 2001; Baykina and Schwarzhans 2017). Three species from Oligocene marine deposits of the Caucasus have been referred to ‘*Pomolobus*’ by Danil’chenko (1960), although this genus is currently considered a junior synonym of *Alosa*. Several diagnostic characters of the Alosinae are evident in the Oligocene species referred to ‘*Pomolobus*,’ but its meristic complement greatly differs from that of the genus *Alosa* (see Table 1), suggesting that additional comparative information would be desirable to conclusively demonstrate their separate generic status. Murray et al. (2005) described a clupeid fish from the Oligocene of Egypt (*Chasmoclupea aegyptica*) and concluded that it may be a member of either the Clupeinae or the Alosinae. The large body size of this fossil and the high and well-developed abdominal scutes support the hypothesis that *Chasmoclupea* can be aligned with the shads. According to Baykina (2015) ‘*Clupea*’ *inflata* from the Miocene of the Balkan Peninsula is likely a member of the Alosinae. The extinct genera *Alisea*, *Ganoessus*, *Xyrinus*, and *Ganolytes* from the Upper Miocene sediments of California (Jordan 1919, 1920, 1921; Jordan and Gilbert 1919) have been referred to the Clupeidae by several authors (e.g., David 1943; Grande 1985; Fierstine et al. 2012). Because of their large size (about 40–60 cm) and their well-developed abdominal scutes, they could tentatively be regarded as alosines; however, additional comparative information would be necessary to definitively verify this assumption. More recently, Baykina and Schwarzhans (2017) performed a revisionary analysis of ‘*Clupea humilis*’ from the Middle Miocene of Moldova and assigned it to a new genus and species (*Moldavichthys switshenskae*) of the Alosinae. Finally, a few species from the Pliocene of Russia have been referred to the extant genus *Hilsa* (see Gabelaya 1976), although their placement appears to be doubtful (Grande 1985).

## Conclusions

The morphological analysis of the near-complete articulated skeleton documented herein has revealed the existence of a new clupeoid taxon in the Eocene limestone of the Pesciara site. The presence of a single specimen of *Eoalosa janvieri* at the Pesciara site is not surprising. Several teleost taxa from Bolca, including the anchovy *Eoengraulis fasoloi* (see Marramà and Carnevale 2016), are represented by a unique known specimen, suggesting that they were adventitious visitors of the Bolca palaeobiotopes. The presence of engraulids, dussumieriines, clupeines, and possibly alosines in the Eocene deposits of Monte Bolca provides evidence of the heterogeneity of clupeid fishes in this celebrated fossil-Lagerstätte, which reflects the outstanding diversity of the entire ichthyofauna (see Carnevale et al. 2014). Because of their abundance, clupeid fishes certainly played an important ecological role in the Pesciara palaeobiotope (Marramà et al. 2016a), which was located in a palaeoenvironmental context close to the coast, and subject to the influence of the open sea (Landini and Sorbini 1996; Marramà et al. 2016a).
